# Night-time heat: How real-world exposure and human diversity redefine vulnerability

**DOI:** 10.1016/j.eehl.2026.100244

**Published:** 2026-04-20

**Authors:** Ursula Debarnot

**Affiliations:** Laboratoire Interuniversitaire de Biologie de la Motricité (LIBM), Université Claude Bernard Lyon 1, Villeurbanne, France

**Heat-health science remains dominated by daytime, outdoor metrics, yet recovery is lost at night and in indoor environments where heat actually accumulates. I argue that heat should be reframed as a 24-h ecological exposure centred on nocturnal recovery and human diversity. Integrating sleep, mental health, metabolism, and sex-specific physiology into multimodal real-world monitoring offers a path to redefine exposure metrics and design fairer, more effective adaptation strategies for a rapidly warming world.**

## 1 Night-time heat: The critical yet missing exposure

Heat is one of the fastest-growing environmental health threats, with global exposure and inequalities sharply rising [1]. Yet most assessments still prioritize daytime outdoor indices, overlooking the phase of the 24-h cycle when vulnerability intensifies [2]. In much of the world, nights are warming faster than days [1,3], while humidity and urban thermal inertia increasingly constrain heat dissipation [[4], [5], [6]]. Temperature–humidity interactions differ physiologically across daytime wakefulness and nighttime sleep, involving distinct thermoregulatory processes [4]. Nighttime therefore constitutes the primary window for psychophysiological recovery, as sleep supports the restoration of key physiological, neural, and emotional functions [3,6,7], all of which depend on adequate nocturnal heat loss, which warm nights increasingly fail to provide [3,5]. Even modest nighttime warming shortens and fragments sleep and disrupts circadian rhythms [3,6,8], and the Lancet Countdown warns that billions of people already lose several nights of sleep annually due to rising temperatures [1].

This loss is not benign. Heat-disrupted sleep weakens physiological and psychological resilience, and reduces tolerance to next-day heat [3,6,7]. Importantly, harm often occurs without reaching classical “dangerous” core temperatures; instead, resilience erodes cumulatively across repeated warm nights [3], a pattern linked to increased mortality under future climate scenarios [8]. Yet this nocturnal erosion of recovery remains largely invisible in environmental health frameworks [1,9,10]. Night-time heat is not a peripheral detail; it is the central blind spot.

## 2 Why current metrics fail to capture real exposure and real people

This blind spot reflects the limits of current metrics, which still frame heat as a meteorological variable rather than as an exposure shaped by built environments, cooling behaviours, psychological states, and the multisystem physiology [4,9,10]. Indoor environments, where people spend most of a heatwave, often remain warmer and more humid than outdoors at night [1,6,9]. While daytime humidity mainly amplifies heat strain by limiting evaporative cooling during activity, nocturnal humidity constrains heat loss under low metabolic conditions, delaying core temperature decline and impairing sleep-dependent autonomic recovery [6,10]. These indoor nocturnal conditions are physiologically consequential yet rarely measured [3,5]. Most mechanistic heat studies rely on short exposures, controlled humidity, minimal radiant load, and homogeneous samples—typically young, healthy men—which limits ecological validity [2,5,11]. As a result, behavioural thermoregulation, cumulative strain, sex-specific physiology, sleep disturbance, and climate diversity remain poorly captured [11,12]. Only a few groups, including the Heat and Health Research Incubator at the University of Sydney, have extended climatic-chamber paradigms toward greater ecological realism, which remains limited in scope.

These constraints matter particularly because human vulnerability is highly heterogeneous. Sex differences shape thermosensation, sweating efficiency, vasodilation, endocrine–circadian coupling, sleep architecture, and thermal discomfort [11,13], and these profiles shift across menstrual and menopausal transitions [11,13]. Mental-health conditions, chronic illness, medication use, and metabolic inflexibility further amplify vulnerability [7,13,14]. While increasingly examined as effect modifiers, these characteristics are often not embedded within the physiological pathways linking heat exposure to impaired recovery, leading to an incomplete characterization of heat-related risk [5,11].

Climate diversity also reshapes physiological load. Hot-humid regions suppress evaporative cooling [4,10], hot-dry climates impose steep diurnal fluctuations [12], and temperate regions increasingly face clusters of warm nights, with pronounced indoor–outdoor divergences during heatwaves [1,9]. Yet tropical populations, nearly half of humanity, remain underrepresented in mechanistic studies despite intense heat–humidity exposure [12].

The translation of climatic stress into health outcomes is further shaped by socioeconomic context and urban development [15]. Temperature–health associations are often attenuated in more affluent and highly urbanised settings despite stronger urban heat island effects, reflecting unequal access to cooling infrastructure, housing quality, healthcare, green space, and social resources [9,15]. This mismatch between exposure, risk distribution, and adaptive capacity underscores the need to integrate socioeconomic context into human diversity frameworks and equitable adaptation strategies [5,15].

## 3 A 24-h ecological framework for heat exposure

Repositioning heat as a 24-h ecological exposure moves beyond current metrics and is essential for understanding how heat interacts with real environments and the psychophysiological processes shaping human vulnerability. In this framework, heat is not a static parameter but a dynamic process unfolding across the day–night cycle through continuous interactions between climate, built environments, behavioural routines, and multisystem psychophysiology [4,5,9]. Vulnerability emerges from the spatiotemporal organisation of exposure and recovery rather than from exposure at a single location and time, including the accumulation of thermal load within buildings, transient micro-exposures during daily mobility between indoor and outdoor environments, behavioural regulation of heat stress, and time-dependent autonomic, metabolic, and cognitive responses [2,6]. This view replaces single-point temperature readings with a model of heat exposure that is relational, cumulative, and biologically consequential [4,5].

Adopting this ecological perspective also changes how heat should be assessed. It requires attending to the mechanisms through which thermal stress becomes embodied: the thermal inertia and ventilation patterns that shape indoor microclimates [1,9], the behavioural adjustments individuals make across the day [4], and the biological diversity that modulates thermal sensitivity and recovery capacity [7,11,13]. These processes are embedded within climatic and cultural contexts that determine humidity patterns [4,10], diurnal variability [12], housing characteristics and routines [9].

Recent advances in multimodal ecological monitoring now make this reconceptualisation operational. Wearables, indoor microclimate sensors and sleep–wake systems can continuously quantify thermal load, physiological regulation, sleep organisation, behavioural thermoregulation, and cognitive–emotional strain in real environments [6,10,14]. By unifying exposure and response within a single ecological paradigm, this framework establishes the foundation for rethinking how heat is measured, modelled and addressed in practice.

## 4 Roadmap for repositioning heat-health science around recovery and diversity

Building on this ecological foundation, heat-health science can now realign measurement and intervention with the realities of lived exposure. The following five priorities translate this conceptual shift into practical action:

Develop 24-h exposure metrics. Exposure metrics must be rebuilt to capture the full thermal experience rather than daytime extremes alone. This includes indoor temperature and humidity, radiant load, cooling behaviour and buildings' thermal inertia [4,6,9], as well as the body's thermal dynamics across contexts. It also requires accounting for short-lived exposures occurring during daily mobility, which contribute to cumulative heat load and behavioural thermoregulation [2]. Widely used indices, such as the Wet-Bulb Globe Temperature, underestimate liveability and survivability thresholds [4,5,12], as they overlook cumulative overnight load, indoor conditions, and the physiological constraints on heat dissipation during sleep. A new generation of metrics should therefore reflect diurnal cycles, indoor–outdoor divergences, thermophysiological responses, and behavioural patterns across space and time. Such metrics can be operationalised within longitudinal multilevel or mediation frameworks linking time-resolved personal thermal load, sleep continuity as a core recovery endpoint, and physiological proxies of heat strain, enabling cumulative and night-specific effects to be quantified without reliance on fixed universal thresholds [4,6].

Make sleep and cognitive–emotional function core endpoints. Because recovery determines next-day resilience, sleep should be treated as a primary environmental health indicator. Heat-disrupted sleep impairs autonomic stability, circadian alignment, emotional regulation, metabolic control, and heat tolerance [3,6,7,13], yet these dimensions remain largely absent from heat-health research. Embedding sleep features, mood, executive function, and cognitive fatigue into exposure frameworks would align heat-health assessment with the physiological and psychological systems that fail first under nocturnal warming.

Embed human diversity into mechanistic and ecological models. Sex, age, hormonal transitions, mental health, metabolic flexibility, physical activity, and chronic disease all shape thermoregulation, sleep, and recovery [7,11,13,14]. Treating these characteristics as covariates systematically underestimates heat-related risk [5,11]. Heat-health models should instead place human diversity at the center of analysis by recognising these factors as determinants of thermoregulatory capacity and sleep-dependent recovery, incorporating sex-specific physiology and sleep responses, age-related vulnerability, mental health, thermal sensitivity, and impaired metabolic regulation under heat. Vulnerability is further modulated by socioeconomic conditions and urban context, making the integration of biological and social dimensions essential for equitable heat-adaptation strategies [15].

Deploy ecological multimodal monitoring. Continuous real-world monitoring of thermal load, sleep–wake dynamics, autonomic responses, cognition, mood, and behavioural thermoregulation can bridge the long-standing gap between physiology and epidemiology [6,10,14]. Wearables, environmental sensors, and indoor microclimate measurements capture how heat accumulates indoors, how cooling behaviour adapts, and how fatigue, mood, and physiological strain evolve across successive warm nights [7]. Scaling such an approach across climates enables the assessment of cumulative strain, behavioural adaptation, and day–night exposure patterns that remain invisible to laboratory or meteorological metrics. Large-scale deployment nonetheless faces practical challenges related to device cost and maintenance, validity under real-world humid heat, participant burden, data integration and governance constraints, and equity-constrained settings, which shape implementation feasibility.

Test adaptation strategies across diverse climatic realities. Cooling strategies, ventilation patterns, sleep-focused interventions, building design, and public health measures must be evaluated across hot-humid, hot-dry, tropical, and temperate contexts [9,12]. The effectiveness and equity of any intervention depend on climate, culture, infrastructure, and physiology [1]. Testing strategies under realistic, behaviourally relevant night-time conditions is essential for identifying which approaches preserve sleep, recovery, and next-day resilience, particularly for populations with heightened vulnerability.

## 5 Conclusion

**Fig. 1:**
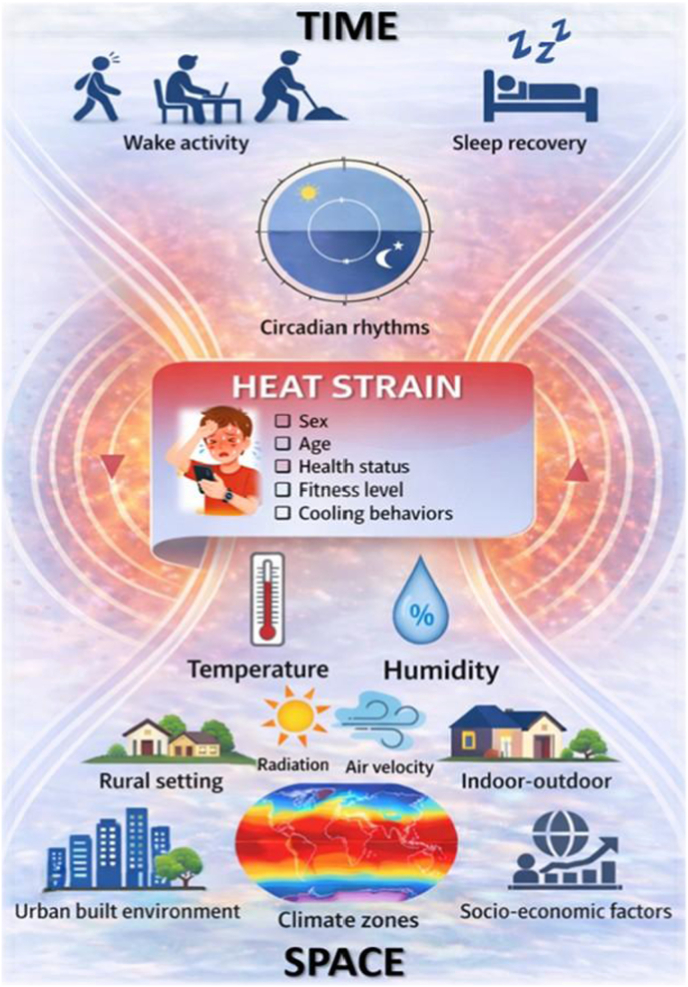
A 24-h ecological framework of heat exposure. Heat exposure is conceptualized as a continuous process unfolding across the day-night cycle and across environments. Environmental drivers, built environments, and individual characteristics interact to shape thermoregulation, sleep recovery, and cumulative heat strain. Multimodal ecological monitoring integrates wearable sensors, indoor microclimate measurements, and smartphone-based assessments to capture real-world exposure and human vulnerability.

Night-time heat is where climate, built environments, behaviour and human biology converge, yet it remains the most physiologically consequential and least measured dimension of exposure. Reframing heat as a 24-h ecological process centred on nocturnal recovery and human diversity provides a renewed scientific and public-health foundation. This integration shifts heat–health assessment from daytime metrics to a 24-h, space–time, individual-centred model of heat strain (Fig. 1). What follows is not simply improved measurement, but the foundation for adaptation strategies that are scientifically robust, locally relevant, and socially equitable, while enabling cross-disciplinary translation between physiology, built environments, and public-health action.

## Declaration of competing interest

The author declares that they have no known competing financial interests or personal relationships that could have appeared to influence the work reported in this paper.
